# A Modeling Approach to Quantify the Effects of Stomatal Behavior and Mesophyll Conductance on Leaf Water Use Efficiency

**DOI:** 10.3389/fpls.2016.00875

**Published:** 2016-06-17

**Authors:** Dany P. Moualeu-Ngangue, Tsu-Wei Chen, Hartmut Stützel

**Affiliations:** ^1^Vegetable Systems Modelling Section, Institute of Horticultural Production Systems, Leibniz Universität HannoverHannover, Germany; ^2^UMR759 Laboratoire d'Ecophysiologie des Plantes sous Stress Environnementaux, Institut National de la Recherche AgronomiqueMontpellier, France

**Keywords:** Light regulation, water use efficiency, sap flow dynamics, stomatal behavior, mesophyll conductance

## Abstract

Water use efficiency (WUE) is considered as a determinant of yield under stress and a component of crop drought resistance. Stomatal behavior regulates both transpiration rate and net assimilation and has been suggested to be crucial for improving crop WUE. In this work, a dynamic model was used to examine the impact of dynamic properties of stomata on WUE. The model includes sub-models of stomatal conductance dynamics, solute accumulation in the mesophyll, mesophyll water content, and water flow to the mesophyll. Using the instantaneous value of stomatal conductance, photosynthesis, and transpiration rate were simulated using a biochemical model and Penman-Monteith equation, respectively. The model was parameterized for a cucumber leaf and model outputs were evaluated using climatic data. Our simulations revealed that WUE was higher on a cloudy than a sunny day. Fast stomatal reaction to light decreased WUE during the period of increasing light (e.g., in the morning) by up to 10.2% and increased WUE during the period of decreasing light (afternoon) by up to 6.25%. Sensitivity of daily WUE to stomatal parameters and mesophyll conductance to CO_2_ was tested for sunny and cloudy days. Increasing mesophyll conductance to CO_2_ was more likely to increase WUE for all climatic conditions (up to 5.5% on the sunny day) than modifications of stomatal reaction speed to light and maximum stomatal conductance.

## Introduction

Stomata on the leaf surfaces of higher plants control the fluxes of gases between the atmosphere and the leaf mesophyll. Regulation of stomata by guard cells determines the amount of CO_2_ available for photosynthesis (*A*) and the amount of water loss through transpiration (*E*; Lawson et al., 2014). To model the relationship between net assimilation rate and stomatal conductance, several approaches have been developed in the literature. A strong correlation between stomatal conductance and net photosynthesis (*A*_net_) is often observed (Wong et al., 1979; Ball et al., 1987) and modeling this relationship has been attempted (e.g., Hari et al., 1986; Lloyd, 1991; Arneth et al., 2002; Katul et al., 2009; Medlyn et al., 2011; among others; see also Damour et al. (2010) for review of stomatal models). Most of the proposed models of stomata only capture the steady state stomatal conductance, which may occur only exceptionally in natural environments. However, a steady state model is not suitable for evaluating continuous changes in *A*_net_ and *E* which define water use efficiency (WUE; *A*_net_/*E*, WUE, Hubick et al., 1986).

WUE is one of the traits considered as determinant of yield under water limited conditions and even as a component of crop drought resistance (Blum, 2009). Several studies have reported considerable variations in WUE among crop species (see Ehdaie et al., 1991 and references therein). For steady state conditions, WUE can be determined by gas exchange measurements. However, fluctuations in climatic conditions lead to changes in stomatal conductance and consequently different instantaneous variations of *E* and *A*_net_. Therefore, WUE varies with fluctuating climate conditions during the course of the day and stomatal behavior. Stomatal behavior is characterized by the speed of aperture/closure and the initial time lag for stomatal response to light stimuli, and related to stomatal morphology, e.g., stomatal size and density (Lawson and Blatt, 2014). For example, modifying stomatal density and stomatal aperture speed might increase chloroplastic CO_2_ concentration (*C*_c_) and therefore WUE (Merlot et al., 2002; Schlüter et al., 2003; Büssis et al., 2006; Lawson and Blatt, 2014). However, the effect of increasing stomatal density on WUE is still difficult to evaluate and it is still unclear if stomatal speed or stomatal density might be more beneficial for WUE because the magnitude of these effects cannot be easily quantified by experiments. Technically, it is difficult to monitor WUE dynamics due to the variation of stomatal conductance to water vapor (*g*_sw_) related to environmental factors. Mathematical modeling of stomatal dynamics was proposed to assess the effects of varying conditions on stomatal behavior (Vialet-Chabrand et al., 2013). The model of Vialet-Chabrand and colleagues includes a steady state target function and parameters quantifying stomatal speed and initial time lag for stomatal response. Replacing the steady state target function by a model of stomatal conductance (e.g., Medlyn et al., 2011) may allow us to quantify the influence of stomatal behavior on *A*_net_ and *E* under fluctuating climatic conditions.

Mesophyll CO_2_ conductance (*g*_m_) is a limiting factor for CO_2_ diffusion into the chloroplast and represents therefore a limitation for net assimilation with a comparable magnitude with stomatal conductance (Flexas et al., 2008). Although the relationship between *g*_m_ and WUE under drought stress is still subject to discussion (Hommel et al., 2014), changes in *g*_m_ were interpreted as adaptation strategy for plants under stress (Warren and Adams, 2006; Aranda et al., 2007). Therefore, genotypes with higher WUE were found to have higher *g*_m_. In contrast to stomatal conductance which influences both water loss and carbon gain, the effect of mesophyll conductance might be predomiantly on *A*_net_, thereby, increasing WUE (Flexas et al., 2008).

The aim of this study was to investigate the influence of stomatal behavior and mesophyll CO_2_ conductance on daily WUE for leaves of a cucumber plant grown under non-stress conditions using a modeling approach. The model includes the Farquhar-von Caemmerer-Berry model of C_3_ photosynthesis (Farquhar et al., 1980), a steady state target stomata model (Medlyn et al., 2011), dynamics of stomatal reactions to environmental stimuli (Vialet-Chabrand et al., 2013), water transport in the leaf (Guyot et al., 2012; Sack and Scoffoni, 2012; Scoffoni et al., 2012; Caldeira et al., 2014; Tardieu et al., 2015), and a transpiration model (Steppe et al., 2006; Jones, 2013). This model was used to test the following hypotheses: (1) faster stomatal regulation results in a higher WUE, (2) higher stomatal density (quantified by increasing maximum stomatal conductance) leads to a decrease of WUE for all light conditions, and (3) higher mesophyll CO_2_ conductance increases WUE.

## Materials and methods

### Model description

Variation in mesophyll water content [*W*_m_(t), mol H_2_O m^−2^] at time *t* is modeled as the difference between water influx [*F*_i_(t), mol H_2_O m^−2^s^−1^] and efflux [*F*_O_(t), mol H_2_O m^−2^s^−1^]:

(1) $$\begin{array}{l} \frac{dW_{\text{m}}}{\;dt}=F_{\text{i}}\left(t\right)-F_{O}\left(t\right). \end{array}$$

At the leaf level, *F_O_*(t) can be assumed to equal the transpiration rate [*E*(t), mol H_2_O m^−2^s^−1^] (Jones, 2013). *F*_i_(t) depends on the difference of water potential between the xylem and the mesophyll (Steppe et al., 2006; Jones, 2013):

(2) $$\begin{array}{l} F_{\text{i}}\left(t\right)=k_{\text{x}}\left(\psi_{\text{x}}-\psi_{\text{m}}\left(t\right)\right), \end{array}$$

where ψ_x_ and ψ_m_(*t*) are the water potentials (MPa) of xylem and mesophyll, respectively, and *k*_x_(mol H_2_O m^−2^s^−1^MPa^−1^) is the xylem and mesophyll, determined by water transport pathways through multiple components, environmental conditions and time of day (Guyot et al., 2012; Sack and Scoffoni, 2012; Scoffoni et al., 2012; Caldeira et al., 2014; Tardieu et al., 2015). For example, the circadian rhythm of hydraulic conductance has a peak in the early morning (Caldeira et al., 2014; Tardieu et al., 2015). The hydraulic conductance *k*_x_ (mol H_2_O m^−2^s^−1^MPa^−1^) is modeled as sum of a radiation dependent and a water potential and oscillation dependent component (Caldeira et al., 2014; Tardieu et al., 2015) by:

(3) $$\begin{array}{l} k_{\text{x}}={cv}_{\text{f}1}*\left(k_{\text{x},0}+k_{\text{x},\text{C}}\right), \end{array}$$

Where

(4) $$\begin{array}{l} k_{\text{x},0}=a+b\;\min\left(\frac{\text{PPFD}}{\text{PPF}{\text{D}}_{\text{lim}}},\;1\right), \end{array}$$

and

(5) $$\begin{array}{l} k_{\text{x},\text{C}}=\tau_{\text{C}}\;\cos\left(-\frac{\pi }{60}t\;+\;\frac{5\pi }{2}\right)*\left(\psi_{\text{x}}-\psi_{\text{m},\text{r}}\right), \end{array}$$

where *k*_x, 0_ (g H_2_O m^−2^s^−1^ MPa^−1^) is the component of xylem hydraulic conductance that depends on irradiance, *a* and *b* are empirical constants (g H_2_O m^−2^s^−1^MPa^−1^). A constant water potential in the xylem sap flow is assumed (ψ_x_ = −0.08 MPa). *k*_x, C_ (g H_2_O m^−2^s^−1^MPa^−1^) is the oscillation dependent component of the hydraulic conductance. τ_C_ = 0.324/15 g H_2_O m^−2^s^−1^ MPa^−2^ is the sensitivity to the amplitude of ψ_x_, *cv*_f1_ = 0.05556 mol g^−1^ H_2_O is the conversion factor from g to mole H_2_O and ψ_m, r_ = −0.9 MPa is the mesophyll reference water potential. *PPFD*_lim_ = 1000 μmol photon m^−2^ s^−1^ is the limit PPFD for the plant leaf. Parameters *a* and *b* were estimated using the literature (Caldeira et al., 2014; Tardieu et al., 2015). Assuming that the matric and gravitational components of the water potential in the leaf are negligible, ψ_m_(t) can be calculated by the contributions of solute and hydrostatic pressure to water potential, ψ_s_ and ψ_p_, respectively:

(6) $$\begin{array}{rcl} \psi_{\text{m}}\left(t\right) & = & \psi_{\text{s}}\left(t\right)+\psi_{\text{p}}\left(t\right) \end{array}$$

(7) $$\begin{array}{rcl} \psi_{\text{s}}\left(t\right) & = & -cv_{\text{f}2}\;RT_{\text{l}}\left(t\right)\frac{N_{\text{m}}\left(t\right)}{W_{\text{m}}}, \end{array}$$

(8) $$\begin{array}{rcl} \psi_{\text{p}}\left(t\right) & = & \alpha e^{-\beta \left(1-\frac{W_{\text{m}}\left(t\right)}{W_{\text{m\_max}}}\right)} \end{array}$$

where *N*_m_ (mol solute) is the total amount of dissolved solutes, *cv*_f2_ = 55.56 mol L^−1^ H_2_O is the conversion factor from liter to mole H_2_O, *R* = 8.3145 10^−3^ L MPa mol^−1^ K^−1^ is the gas constant and *T*_l_(t) is the leaf temperature at time t (in K). *W*_m_(t) is the water content in the mesophyll cell at time *t* (mol H_2_O m^−2^). The relationship between hydrostatic pressure and relative cell volume *W*_m_(t)/*W*_m_max_ is an approximation deduced from Steudle et al. (1977), where *W*_m_max_ is the maximum water content of the mesophyll, *W*_m_(t) is the water content in the mesophyll cell at time *t*, α (MPa) is the full hydrostatic pressure and ß is a measure of mesophyll elasticity.

Variation of the total amount of dissolved solutes in the mesophyll is given by:

(9) $$\begin{array}{l} \frac{dN_{\text{m}}}{dt}=c_{\text{i}}\;N_{\text{xy}}F_{\text{i}}\left(t\right), \end{array}$$

where *c*_i_ (unit-less) is a factor for ion exchange, and *N*_xy_ denotes the solute concentration in the xylem sap (mol solute mol^−1^ H_2_O).

The transpiration rate [*F*_*O*_(t), mol m^−2^s^−1^] is modeled as a function of leaf temperature (Maes and Steppe, 2012; Jones, 2013; Tardieu et al., 2015):

(10) $$\begin{array}{l} F_{\text{O}}\left(t\right)=\frac{1}{r_{\text{tw}}}\frac{\rho_{\text{a}}c_{\text{p}}\left(\delta e+s\;\left(T_{\text{l}}\left(t\right)-T_{\text{a}}\right)\right)}{\lambda \gamma }, \end{array}$$

where ρ_a_ = 1.205 10^3^ is air density (g m^−3^), *c*_p_ = 1.005 the heat or thermal capacity of air (J g^−1^ K^−1^), *s* is the slope of the curve relating temperature to saturated vapor pressure (kPa K^−1^), *T*_a_ is air temperature (*K*), λ = 4417 J m^−3^ is the latent heat of water vaporization, γ = 0.0665 kPa K^−1^ is the psychrometric constant. *r*_tw_ = 1/g_tw_(t) is the total resistance to water vapor transport (s m^−2^mol^−1^H_2_O) and [*g*_tw_(t)] is the total conductance to water vapor transport (mol H_2_O m^−2^s^−1^):

(11) $$\begin{array}{l} g_{\text{tw}}\left(t\right)=\frac{g_{\text{bw}}g_{\text{sw}}\left(t\right)}{g_{\text{sw}}\left(t\right)+g_{\text{bw}}}; \end{array}$$

where *g*_bw_ is the boundary layer conductance (mol H_2_O m^−2^s^−1^) and *g*_sw_ the stomatal conductance to water vapor. δ*e* is the vapor pressure deficit (kPa) defined by:

(12) $$\begin{array}{l} \delta e=\left(1-\frac{h_{\text{r}}}{100}\right)a_{1}\;\exp\;\left(\frac{a_{2}\left(T_{\text{a}}-273.16\right)}{a_{3}+\left(T_{\text{a}}-273.16\right)}\right) \end{array}$$

where *h*_r_ is the relative humidity of the ambient air (%), *a*_1_ = 0.61375 kPa*, a*_2_ = 17.502, and *a*_3_ = 240°C. The value of −273.16 is required for the conversion from °K to °Celsius. The slope of the curve relating saturated vapor pressure to temperature is therefore defined by:

(13) $$\begin{array}{l} s=\frac{a_{2}a_{3}\delta e}{{\left(a_{3}+T_{\text{a}}-273.16\right)}^{2}}. \end{array}$$

According to Maes and Steppe (2012) the relation between (*T*_l_ − *T*_a_) and *r*_*aH*_ = 1/*g*_*aH*_, the resistance to diffusive heat transfer to air, is given by:

(14) $$\begin{array}{l} T_{\text{l}}-T_{a}=\frac{r_{\text{aH}}r_{\text{tw}}\gamma \left(R_{\text{n}}-G_{i}\right)-{r_{\text{aH}}\rho }_{\text{a}}c_{\text{p}}\delta e}{\rho_{a}c_{p}\left(\gamma r_{\text{tw}}+sr_{\text{aH}}\right)}, \end{array}$$

where *R*_n_ (J m^−2^s^−1^) is radiation and *G*_i_ (J m^−2^s^−1^) is soil heat flux which is here assumed to be zero since an individual leaf is considered. The stomatal conductance to water vapor is assumed to be 1.6 times the stomatal conductance to CO_2_ as usually used in the literature (Medlyn et al., 2002, 2011) where 1.6 is the ratio of the diffusivities of CO_2_ and water in air. The conductance to diffusive heat transfer to air is related to the boundary layer conductance through the relation *g*_aH_ = *g*_bH_*/1.15* where 1.15 is the product of the ratio of the diffusivities of heat and water in the boundary layer (dimensionless). Vialet-Chabrand et al. (2013) proposed a dynamic model to describe the temporal response of stomatal conductance to water vapor, denoted by *g*_sw_ (mol H_2_O m^−2^ s^−1^) to a change of irradiance over time:

(15) $$\begin{array}{l} \frac{dg_{\text{sw}}}{dt}=\alpha_{\text{g}}\;\left(\text{ln}\left(\frac{1.6G\left(t\right)-r_{0}}{g_{\text{sw}}\left(t\right)-r_{0}}\right)\right)\left(g_{sw}\left(t\right)-r_{0}\right) \end{array}$$

where *r*_0_ (mol m^−2^ s^−1^) is a parameter describing the initial time lag of *g*_sw_ after exposure to an environmental stimulus, α_g_ is a time constant (s^−1^) for increasing or decreasing of *g*_sw_ and *G* is the steady-state target value of stomatal conductance to CO_2_ under the current environmental conditions described by Medlyn et al. (2011) and Chen et al. (2014). Incorporating stomatal response to leaf water potential as presented by Tuzet et al. (2003), the steady state target stomatal conductance *G* is defined by:

(16) $$\begin{array}{l} G=g_{0}+\left(1+\frac{g_{1}}{\sqrt{\delta e}}\right)\frac{A}{C_{\text{a}}}f_{\psi_{\text{m}}}=\;g_{0}+g\text{s}{\text{c}}_{\text{b}}A \end{array}$$

where parameters *g*_0_ (mol CO_2_ m^−2^s^−1^) and *g*_1_ are species-specific constants of stomatal conductance (for cucumber, 0.009 mol CO_2_ m^−2^s^−1^, and 3.51, respectively, see Chen et al., 2014), *C*_a_ is the ambient CO_2_ concentration at the leaf surface and *f*_ψm_ quantifies the dependency of *G* to mesophyll water potential (Tuzet et al., 2003). *f*_ψm_ is defined by:

(17) $$f_{\psi \text{m}}=\frac{1+\exp\left(s_{\text{f}}\psi_{\text{r}}\right)}{1+\text{exp}(s_{\text{f}}(\psi_{\text{r}}-\psi_{\text{m}})},$$

ψ_r_ = −0.9 MPa is the reference water potential, *s*_f_ = 4.9 MPa^−1^ is an empirical sensitivity parameter of the stomatal reaction to water potential*, A* (μmol CO_2_ m^−2^s^−1^) is the steady-state net photosynthesis rate, i. e., the minimum of the Rubisco-limited (*A*_c_, μmol CO_2_ m^−2^s^−1^) or RuPB-regeneration-limited (*A*_j_, μmol CO_2_ m^−2^s^−1^) photosynthesis rate (Farquhar et al., 1980):

(18) $$\begin{array}{r} A_{c}=\frac{V_{\text{cmax}}\cdot \left(C_{\text{c}}-\Gamma_{*}\right)}{C_{\text{c}}+K_{\text{m}}}, \end{array}$$

(19) $$\begin{array}{r} A_{\text{j}}=\frac{J\cdot \left(C_{\text{c}}-\Gamma_{*}\right)}{4C_{\text{c}}+8\Gamma_{*}}. \end{array}$$

Here, Γ_*_ is the CO_2_ compensation point in the absence of dark respiration (43.02 μmol CO_2_ m^−2^s^−1^), *R*_d_ is the daytime respiration rate (μmol CO_2_ m^−2^s^−1^), *V*_cmax_ (μmol CO_2_ m^−2^s^−1^) is the maximum rate of Rubisco activity at the site of carboxylation, and *K*_m_ is the effective Michaelis-Menten constant for CO_2_ assimilation that considers the competitive inhibition by O_2_ (711 μmol mol^−1^); *J* (μmol e^−^m^−2^s^−1^) is the rate of electron transport and *C*_*c*_ (μmol mol^−1^) is the mole fraction of CO_2_, which is calculated by:

(20) $$\begin{array}{l} C_{\text{c}}\left(t\right)=C_{\text{a}}-A_{c}\frac{G+g_{\text{m}}}{Gg_{\text{m}}} \end{array}$$

where *g*_m_ is mesophyll CO_2_ conductance (mol CO_2_ m^−2^s^−1^) and *C*_*a*_ is the ambient CO_2_ concentration (380 μmol mol^−1^). Therefore, the values of *A*_c_ and *G* are the analytical solutions which satisfy Equations (18–20, 16) at the same time. Replacing *C*_c_ from Equation (20) in Equation (18) yields,

(21) $$\begin{array}{l} A_{\text{c}}\left(t\right)=\frac{V_{\text{cmax}}\cdot \left(C_{a}-A_{\text{c}}\frac{G\left(t\right)+g_{\text{m}}}{G\left(t\right)g_{\text{m}}}-\Gamma_{*}\right)}{C_{\text{a}}-A_{\text{c}}\frac{G\left(t\right)+g_{\text{m}}}{G\left(t\right)g_{\text{m}}}+K_{\text{m}}}. \end{array}$$

As shown by Ögren and Evans (1993), the photosystem II electron transport rate that is used for CO_2_ fixation and photorespiration, *J*, is related to the amount of incident photosynthetically active irradiance (*I*_inc_; μmol photons m^−2^s^−1^) by:

(22) $$\begin{array}{l} J=\frac{\kappa_{2\text{LL}}I_{\text{inc}}+J_{\text{max}}-\sqrt{{\left(\kappa_{2\text{LL}}I_{\text{inc}}+J_{\text{max}}\right)}^{2}-4\theta \kappa_{2\text{LL}}I_{i\text{nc}}J_{\text{max}}}}{2\;\theta }, \end{array}$$

where *J*_max_ (μmol e^−^ m^−2^s^−1^) is the maximum electron transport rate at saturating light levels, θ is a dimensionless convexity factor for the response of *J* to *I*_inc_, and κ_2LL_ (μmol e^−^ m^−2^s^−1^) is the conversion efficiency of *I*_inc_ to *J* at limiting light.

Because *C*_a_, Γ^*^, and *K*_m_ are constant, for simplification we set $p_{1}=C_{\text{a}}-\Gamma^{*}$ and p_2_ = *C*_a_ + *K*_m_. For the sake of simplicity, we also set *g*_0m_ = *g*_0_+*g*_m_. Using Equations (18, 20), the steady-state net photosynthesis rate is the solution of the following equation:

(23) $$\begin{array}{l} c_{3}A_{c}^{3}+c_{2}A_{c}^{2}+c_{1}A_{c}+c_{0}\;=\;0, \end{array}$$

where

$$\begin{array}{lll} c_{3} & = & g\text{s}{\text{c}}_{\text{b}},\\ c_{2} & = & g_{0m}-g\text{s}{\text{c}}_{\text{b}}{\text{V}}_{\text{cmax}}-g\text{s}{\text{c}}_{\text{b}}g_{\text{m}}p_{2},\\ c_{1} & = & g_{\text{m}}p_{1}V_{\text{cmax}}g\text{s}{\text{c}}_{\text{b}}-g_{0m}V_{\text{cmax}}-g_{0}g_{\text{m}}p_{2},\\ c_{0} & = & g_{0}g_{\text{m}}p_{1}V_{\text{cmax}}. \end{array}$$

The RuPB-regeneration-limited photosynthesis rate *A*_j_ can be found combining Equations (16, 19, 20), and rearranging the expressions using previous notations and *k*_1_ = *C*_a_ −Γ^*^ and $k_{2}=C_{\text{a}}+2\Gamma^{*}$, it follows that *A*_j_ satisfies the following equation:

(24) $$\begin{array}{l} b_{3}A_{j}^{3}+b_{2}A_{j}^{2}+b_{1}A_{j}+b_{0}\;=\;0, \end{array}$$

where

$$\begin{array}{lll} b_{3} & = & 4gsc_{b},\\ b_{2} & = & 4g_{0\text{m}}-Jg\text{s}{\text{c}}_{\text{b}}-4k_{2}g_{\text{m}}g\text{s}{\text{c}}_{\text{b}},\\ b_{1} & = & k_{1}g_{\text{m}}Jg\text{s}{\text{c}}_{\text{b}}-4k_{2}g_{\text{m}}g_{0}-Jg_{0\text{m}},\\ b_{0} & = & k_{1}g_{\text{m}}Jg_{0}. \end{array}$$

Equations (23, 24) are solved simultaneously in order to determine the value of *A*. The target photosynthesis rate *A* always exists since there is always at least one real solution of Equations (23, 24). The current photosynthesis rate is determined from the current stomatal conductance.

### Plant materials for model evaluation

Cucumber seeds (*Cucumis sativus*, cv. Aramon, Rijk Zwaan, De Lier, Netherlands) were sown on 10 June 2014 in rock-wool cubes (36 × 36 × 40 mm) in the greenhouse of the Institute of Horticultural Production Systems, Leibniz Universität Hannover, Germany (52.5°N, 9.7°E). Seven days after sowing, seedlings were transplanted into larger rock-wool cubes (10 × 10 × 6.5 cm) for another 7 days. Plants were cultivated on rock wool slabs (Grodan, Grodania A/S, Hedehusene, Denmark), which were placed on metal gutters. The day/night temperature for heating was set to 22°C day/20°C night. Ventilations opened at 24°C during daytime. Each liter of nutrient solution contained 0.5 g Ferty Basisdünger 2 (Planta GmbH, Regenstauf, Germany, 0.9 mM ${\text{NO}}_{3}^{-}$, 1.5 mM ${\text{NH}}_{4}^{-}$, 2.8 mM K^+^, 3.0 mM Ca^2+^, 0.4 mM Mg^2+^, 0.4 mM H_2_PO_4_, as well as adequate amounts of the micronutrients) and extra 0.9 g Ca(NO_3_)_2_ was added in the solution (5.5 mM Ca^2+^ and 11 mM ${\text{NO}}_{3}^{-}$) after the first fruit set.

### Measurements

Sap flow data were collected between 30 and 31 June for model evaluation and between 06 and 07 July 2014 for model validation on a fully expanded leaf of a well-watered cucumber plant. The leaf was located at the upper canopy, positioned toward south-east and not shaded by other leaves. A heat field deformation sensor (HFD) was installed on the petiole of the leaf to monitor the relative water flow through the petiole (Hanssens et al., 2013, 2014) on these days. A quantum sensor (Li190, Li-Cor, Lincoln, USA) of photosynthetically active radiation (PAR) was installed next to the measured leaf in order to capture the PAR intercepted by the leaf. Data of HFD and the quantum sensor were logged every minute. Average air temperature and relative humidity of 12 min in the greenhouse were recorded by sensors installed 2 m above ground, about 2 m from the measured leaf. Leaf temperatures were measured hourly by an infrared camera (E60, FLIR Systems INC, Boston, USA). Leaf water potential (C52-chambers, WESCOR INC, South Logan., USA) and water content were measured every 2 h from 8h00 to 18h00. Since the measurements of water potential and water content were destructive, they were taken on four leaves comparable (leaf age, position, and orientation in the canopy) to the monitored leaf.

### Input data

**Figure 1:**
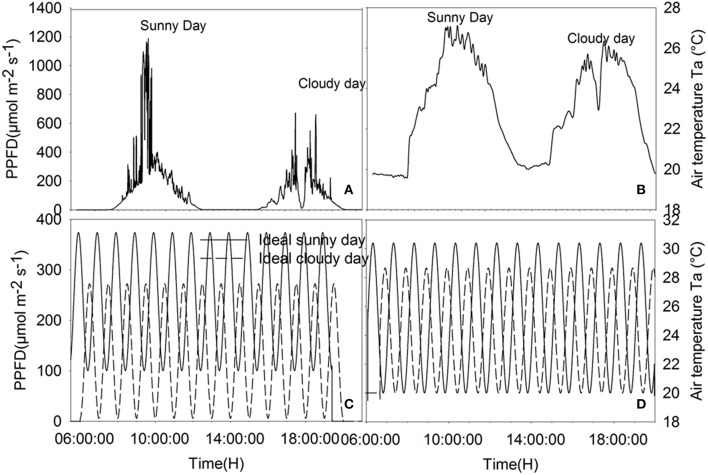
**Climatic data of the cloudy, sunny, ideal cloudy, and ideal sunny days**. **(A)** PPFD recorded on 06-07/08/2014, **(B)** temperature data of recorded on 06-07/08/2014, **(C)** PPFD on ideal sunny and cloudy days, **(D)** ambient temperature on ideal cloudy and sunny days. The integral of total radiation and temperature during the sunny and cloudy days are, respectively, equal to the integral of radiation and temperature during the ideal sunny and cloudy days for a solar period of 2, 1 h, 30 and 15 min.

Climatic data recorded on the 6 and 7 of August 2014, representing a sunny and a cloudy day, respectively (Figure 1), were used as input data. Moreover, to test the effect of rhythmic fluctuation of climatic conditions on WUE, so called “ideal” sunny and cloudy days were simulated. Variation of temperature and radiation during the ideal days was modeled using sinusoidal functions based on Kimball and Bellamy (1986) light and temperature models. The equation defining the light intensity on ideal days is given by:

(25) $$\begin{array}{l} I\left(t\right)=I_{\text{m}}+I_{a}\cos\;\left(\frac{\pi \left(t-t_{0}\right)}{p}-\pi \right), \end{array}$$

where *I*_m_ is the average light intensity during daytime (139 μmol photon m^−2^ s^−1^ on the cloudy day, 237 μmol photon m^−2^ s^−1^ on the sunny day), *I*_*a*_ (μmol photon m^−2^ s^−1^) is the amplitude of the oscillation in light intensity (133.435 on the cloudy day, 137.02 on the sunny day), *p* is the light fluctuation period and *t*_0_ is used to set the initial time point to minimum for the integration. Temperature equations were defined in a similar way and a constant lag of 30 min (observed from recorded data) was kept between light variation and subsequent temperature change. Parameters of the ideal sunny and cloudy days were chosen to ensure that the integrals of radiation and temperature during the sunny day and the cloudy day were equal to the integrals of radiation and temperature on ideal sunny and cloudy days (Figure 1). Using measured data of 06–07/08/2014, a hyperbolic decay function was fitted (*r*^2^ = 0.53, *P* < 0.0001) to describe the relationship between relative humidity and temperature:

(26) $$\begin{array}{l} h_{\text{r}}\left(t\right)=\frac{1173.613}{T_{\text{a}}\left(t\right)-6.3458}, \end{array}$$

which is valid for *T*_a_ > 6.3458.

Parameters of the sinusoidal light and temperature models were fitted so that the integrals of radiation and temperature during the sunny and cloudy days were equal to the integrals of radiation and temperature recorded on 6 and 7/08/2014, respectively. Moreover, simulations were also performed with different sinusoidal time periods to estimate the effects of fluctuation frequencies on WUE.

### Parameter fitting

The stomata model was parameterized using measurements with the Li6400-XT portable photosynthesis system (Li-Cor, Lincoln, USA). The leaf was left in darkness for about 30 min, until stomatal conductance was close to zero. Then, PAR was set to 1300 μmol photon m^−2^ s^−1^ and values of stomatal conductance were logged every 5 s until reaching stability. With these data, the time constant α_*g*_ and the initial lag *r*_0_ were estimated using Equation (15). Using data of 30–31 July 2014, the limit light intensity for conductivity PPFD_lim_ = 1000 μmol photon m^−2^ s^−1^ and the reference mesophyll water potential ψ_m, r_ = −0.9 MPa were estimated to agree with the observations. The ideal light and temperature model parameters representing the average and the amplitude of light and temperature per oscillation period were fitted using recorded light and temperature data in Excel. The relationship between *h*_*r*_ and *T*_*a*_ was fitted using the software Sigmaplot (version 11.0, Systat software GmbH, Erkrath, Germany).

### Sensitivity analysis

To test our hypotheses, sensitivity of WUE to the time constant for stomata aperture and closure, α_g_, initial lag for stomatal reaction, *r*_0_, maximum stomatal conductance (modeled in *g*_1_) and mesophyll CO_2_ conductance were analyzed. Stomatal behavior parameters were fixed to estimated values for cucumber leaves (α_g_=4.0516 10^−3^ s^−1^, *r*_0_ = 2.674 10^−3^ mol H_2_O m^−2^ s^−1^). Then, *r*_0_, was changed to 0.0106, 0.0012, and 0.0002 mol H_2_O m^−2^ s^−1^ and α_g_ was changed decreasingly to 0.00682, 0.00338, 0.00134, and 0.00109 s^−1^ (values in range of the estimates in Vialet-Chabrand et al. (2013)). In each of these scenarios, WUE was computed per day and per 5 s. The daily WUE was computed as net assimilation integral divided by the transpiration integral in the period from 6:00 a.m. to 20:00 p.m. The maximum stomatal conductance, which should be proportional to stomatal density, increases with *g*_1_ in Equation (16). We changed the value of *g*_1_ from −40 to 90% and analyzed the impact on the daily WUE. Mesophyll CO_2_ conductance (*g*_m_ = 0.3 mol CO_2_ m^−2^s^−1^) was increased from −40 to a 500% to evaluate the effects of *g*_m_ on WUE.

“Ideal” light and temperature models (Kimball and Bellamy, 1986) were chosen to represent slow and fast light changing scenarios. Ideal day light intensity was defined with sinusoidal fluctuating functions (day length = 15 h, frequency = 2 h^−1^, 1 h^−1^, 30 and 15 min^−1^).

## Results

**Figure 2:**
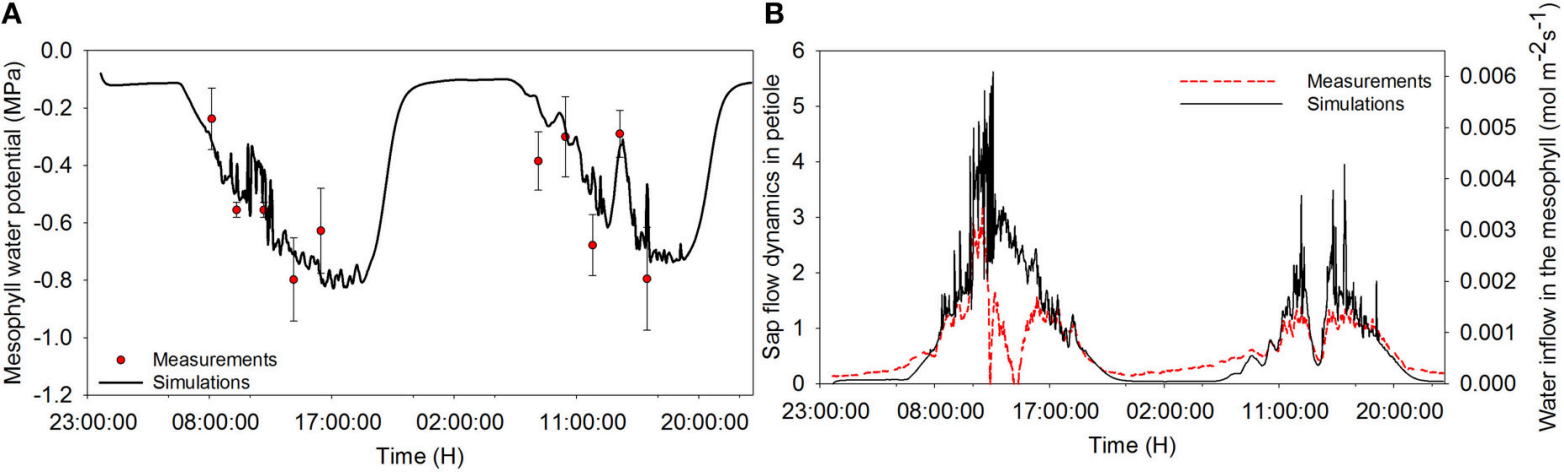
**Diurnal time courses of mesophyll water potential (A) and water inflow to the mesophyll (B) and comparison between simulated and measured data during a cloudy (first day) and a sunny day (second day)**. Dots represent measured data of water potential, and dashed lines represent the sap flow raw data and solid lines are simulations from the model. Climatic data are the two days measurement data presented in Figure 1, and all parameter values are defined in Tables 1, 2.

The model describes well the decreasing trend of relative water content during daytime for both the sunny day and the cloudy day (Figure 2). During daytime, the relative water content (RWC) dropped down to 83% on the sunny day and remained at around 92% during the dark period. The diurnal trend of leaf water potential shows a decreasing trend of water potential during the day. The diurnal course of RWC was well reproduced during the first day (data not shown). On the second day, however, a discrepancy was found between observations and estimation in the morning. Overall, a linear relationship (*r*^2^ = 0.54, *P* = 0.0156) was found between observations and simulations. Observed leaf temperature was higher than the simulated values and no linear relationship between observations and simulations was found (*r*^2^ = 0.01, *P* = 0.641; data not shown).

**Table 1:** **Input and output variables of the model**.

**Variable**	**Description**	**Type**	**Units**	**Equations**	**Initial value**
*F*_*i*_(*t*)	Water flux into the mesophyll	Output	mol H_2_O m^−2^ s^−1^	(2)	
*k*_*x*_(*t*)	Hydraulic conductivity between xylem and mesophyll	Output	mol H_2_O m^−2^ s^−1^MPa^−1^	(3)	
*k*_*x*, 0_	Irradiance dependent component of xylem hydraulic conductance	Input	g H_2_O m^−2^ s^−1^MPa^−1^	(4)	
*k*_*x, C*_	Oscillation dependent component of xylem hydraulic conductance	Input	g H_2_O m^−2^s^−1^MPa^−1^	(5)	
ψ_m_(*t*)	Water potential in the mesophyll	Output	MPa	(6)	−0.08
ψ_*s*_(*t*)	Osmotic potential in the mesophyll	Output	MPa	(7)	−1.28
ψ_p_(*t*)	Hydrostatic pressure in the mesophyll	Output	MPa	(8)	1.2
*W*_m_(*t*)	Water content in the mesophyll cell	Output	mol H_2_O m^−2^	(1)	16
*N*_m_(*t*)	Amount of solute in the mesophyll	Output	mol solute m^−2^	(9)	0.121
*F*_0_(*t*),	Efflux from the mesophyll	Output	mol H_2_O m^−2^s^−1^	(10)	
*E(t)*	Evapotranspiration rate	Output	mol H_2_O m^−2^s^−1^	(10)	
*g*_tw_(*t*)	Total conductance to water vapor transport	Output	mol H_2_O m^−2^s^−1^	(11)	
δ*e*(t)	Vapor pressure deficit	Input	kPa	(12)	
*s*(*t*)	Slope of the curve relating the temperature to the vapor pressure deficit	Input	Pa K^−1^	(13)	
*T*_*l*_(*t*)	Leaf temperature at time *t*	Output	K	(14)	295.15
T_a_ (t)	Air temperature	Input	K	–	
*g*_sw_(*t*)	Stomatal conductance to water vapor	Output	mol H_2_O m^−2^s^−1^	(15)	0.02
*g*_sc_(*t*)	Stomatal conductance to CO_2_	Output	mol CO_2_ m^−2^s^−1^	(1.16)	0.0125
*R*_n_(*t*)	Net radiation	Input	J m^−2^s^−1^	Measured	
*G(t)*	Steady-state target under the current environmental condition	Output	mol CO_2_ m^−2^s^−1^	(16)	
*A(t)*	Steady-state net photosynthesis rate	Output	μmol CO2 m^−2^ s^−1^	(18, 21, 23, 19, 24)	
C_c_ (t)	Chloroplastic CO_2_ concentration	Output	μmol mol^−1^	(20)	
*h*_r_(*t*)	Relative humidity	Input	–	Measured	
*J*(*t*)	Rate of electron transport	Output	μmol e− m^−2^ s^−1^	(22)	
*I*_inc_(*t*)	Amount of incident photosynthetically active irradiance	Input	μmol photon m^−2^ s^−1^	Measured	

**Table 2:** **Model parameters description and estimated values**.

**Parameters**	**Description**	**Unit**	**Value**
*N*_xy_	Solute concentration in the xylem sap	mol mol^−1^ H_2_O	0.0003
*c*_e_	Na^+^ exclusion coefficient	–	0.2
*a*	Empirical constants relating hydraulic conductivity to the mesophyll water potential	g H_2_O m^−2^s^−1^MPa^−1^	0.0259
*b*	Empirical constants relating hydraulic conductivity to the mesophyll water potential	g H_2_O m^−2^s^−1^Mpa^−1^	0.2268
*cv*_f1_	Conversion factor from g to mole H_2_O	mol g^−1^ H_2_O	0.05556
*cv*_f2_	Conversion factor from L to mole H_2_O	mol L^−1^ H_2_O	55.56
ψ_m, r_	Mesophyll reference water potential	MPa	−0.9
*PPFD*_lim_	Limit PPFD for the plant leaf	μmol photon m^−2^ s^−1^	1000
τ_*C*_	Sensitivity to the amplitude	g H_2_O m^−2^s^−1^MPa^−2^	0.324/15
*W*_m_max_	Maximum water content of the mesophyll	mol H_2_O m^−2^	16
ψ_x_	Water potential in the xylem	Mpa	−0.08
*R*	Ideal gas constant	L Mpa mol^−1^ K^−1^	8.314.10^−3^
α	Full turgor pressure	Mpa	2.4
β	Measure of the mesophyll elasticity	–	10.6
ρ_a_	Air density	g mol^−1^	28.9645
*c*_i_	Ion exclusion factor	–	2
*c*_p_	Thermal capacity of the air	J g^−1^ K^−1^	1.012
γ	Psychrometric constant	kPa K^−1^	0.0665
*g*_aW_	Boundary layer Conductance to water transport	mol m^−2^s^−1^	2.7
*g*_aH_	Conductance to sensible heat transport	mol m^−2^s^−1^	*g*_*bw*_/1.15
*a*_1_	Empirical constants relating vapor pressure deficit to relative humidity	kPa	0.61375
*a*_2_	Empirical constants relating vapor pressure deficit to the air temperature	–	17.502
*a*_3_	Empirical constants relating vapor pressure deficit to the air temperature	K	240.97
λ	Latent heat of water vaporization	J mol^−1^H_2_O	44172
*G*_i_	Soil heat storage	J m^−2^s^−1^	0
α_*g*_	Time constant for the stomatal conductance	s^−1^	0.0040516
*r*_0_	Parameter describing the initial time lag	mol H_2_O m^−2^s^−1^	0.002674
*g*_0_	Species-specific constants of stomatal conductance	mol m^−2^s^−1^	0.009
*g*_1_	Species-specific constants of stomatal conductance		3.51
Γ_*_	Constant CO_2_ compensation point of assimilation in the absence of dark respiration	μmol CO_2_ m^−2^s^−1^	43.02
*V*_cmax_	Maximum rate of Rubisco activity at the site of carboxylation	μmol CO_2_ m^−2^s^−1^	102
C_a_	Ambient CO_2_ concentration at the leaf surface	μmol CO_2_ m^−2^s^−1^	380
*R*_*d*_	Dark respiration rate	mol CO_2_ m^−2^s^−1^	1.08
*K*_m_	Michaelis-Menten constants of Rubisco for CO_2_	μmol CO_2_ mol^−1^	711
*g*_m_	Mesophyll CO_2_ conductance	mol CO_2_ m^−2^s^−1^	0.3
*J*_max_	Maximal rate of electron transport	μmol e^−^ m^−2^s^−1^	140
θ	Convexity factor for the response of *J* to *I*_inc_	–	0.75
κ_2LL_	Conversion efficiency of *I*_inc_ into *J* at low light	μmol e^−^ m^−2^s^−1^	0.425

**Figure 3:**
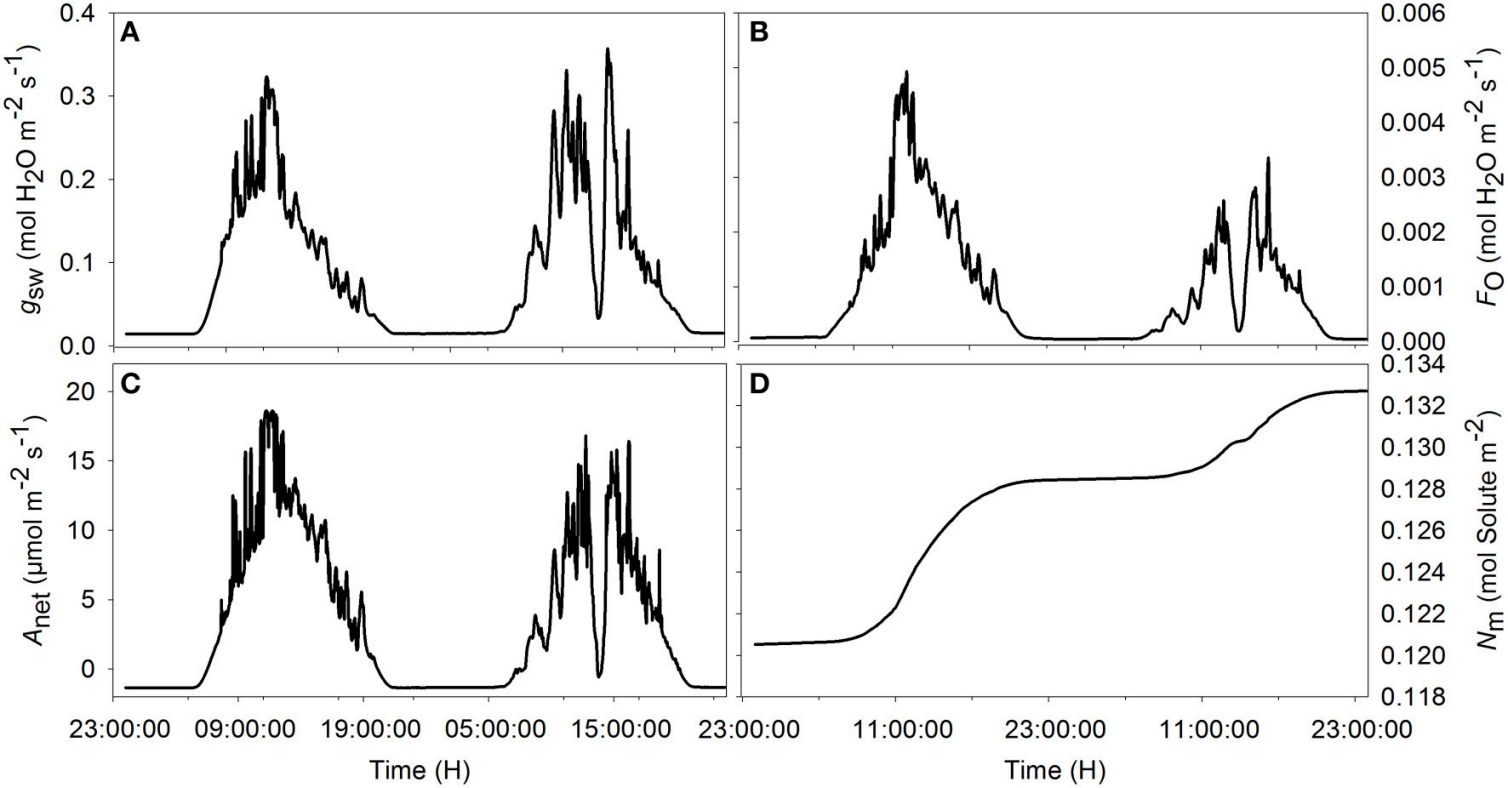
**Simulated diurnal time courses of net assimilation *A*_net_ (A), stomatal conductance to water vapor *g*_sw_ (B), transpiration F_O_ (C), and amount of solute in the mesophyll (D) during the sunny (first day) and cloudy day (second day)**. Climatic data used are from the 2 days of measurement data presented in Figure 1, and all parameter values are defined in Tables 1, 2.

Simulations agreed with the observed trend during both the sunny and the cloudy day. Observed raw sap flow data and simulated water inflow in the mesophyll were correlated with *r*^2^ = 56%. All fluctuations observed in the sap flow dynamics data were reproduced by the model and the delay observed with respect to irradiance was mimicked, although a discrepancy was observed between simulated and observed sap flow dynamics during the sunny day. As expected, the simulated net assimilation rate, the transpiration rate and stomatal conductance followed the course of the sun (Figures 3A–C). Due to the decrease of ψ_m_ during daytime, solute accumulation in the leaf increased during daytime and stabilized during the night (Figure 3D). The model showed a decreasing trend of relative water content during daytime due to transpiration in the presence of irradiance and the circadian clock effects on xylem hydraulic conductivity (data not shown).

### Effects of stomatal speed on WUE

**Figure 4:**
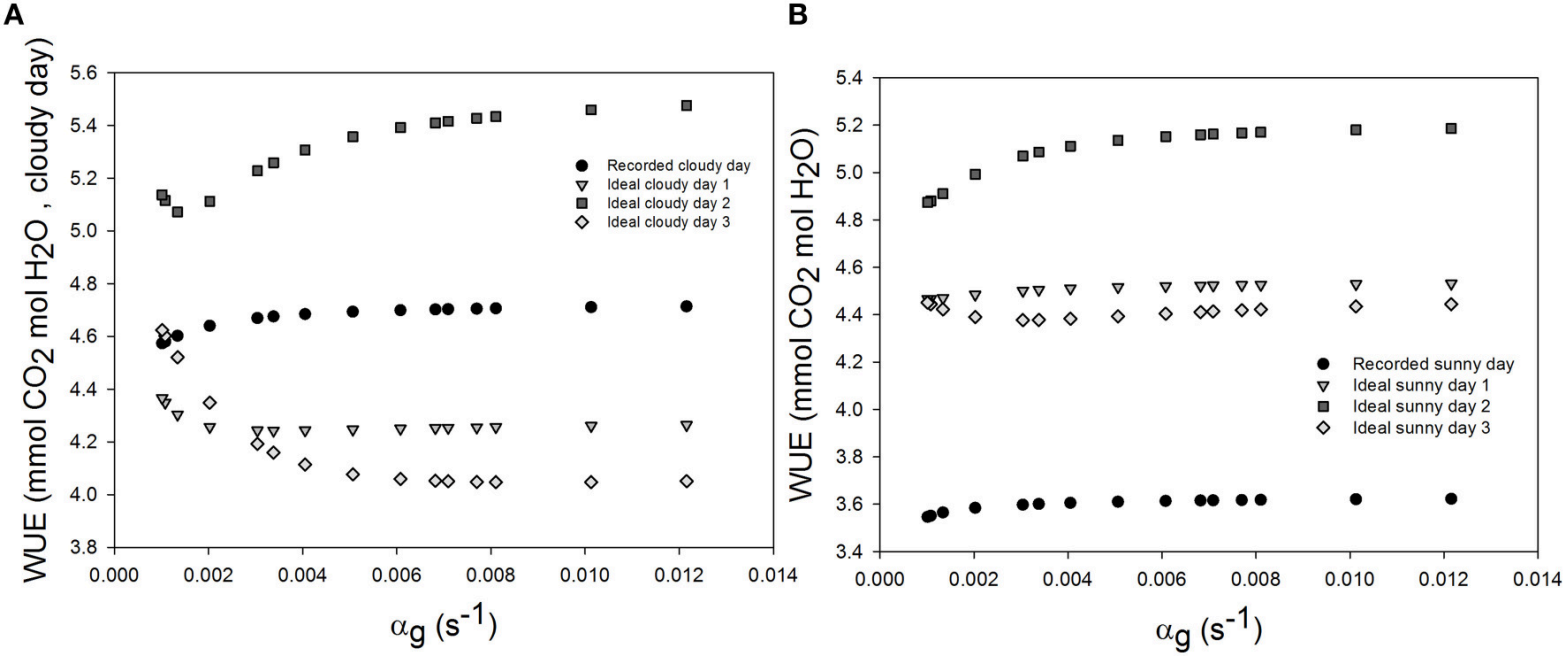
**Effect of varying the time constant for stomata response (α_*g*_) on daily water use efficiency (WUE) during the sunny day (B), the cloudy day (A), and different ideal sunny (B) and cloudy days (A) scenarios**. Simulations were performed per second over 15 h (from 06:00 a.m. to 9:00 p.m.). The speed of stomatal aperture was varied in a set of 15 values and all climatic data were extracted from the 2 days measurement data presented in Figure 1 and ideal sunny and cloudy days scenario. The ideal sunny and cloudy days 1 to 3 corresponds to sinusoidal fluctuation frequency equal to 2 h^−1^, 1 h^−1^ and 30 min^−1^. All other parameter values are defined in Tables 1, 2.

Daily WUE was 3.605 and 4.685 mmol CO_2_ mol^−1^ H_2_O and on the sunny and cloudy day, respectively. Initial time lag for stomatal response did not lead to an appreciable change of daily WUE (<0.5%, figure not shown). Increasing the time constant for stomatal response slightly increased WUE in all scenarios (Figure 4). During the cloudy day, an increase of 1.2% in WUE was reached for an increasing stomatal speed by 60% and a light period of 2 h (Figure 4A). The daily WUE increased by 1.46% for the ideal sunny day with a 2 h period of light and temperature fluctuation (Figure 4B).

**Figure 5:**
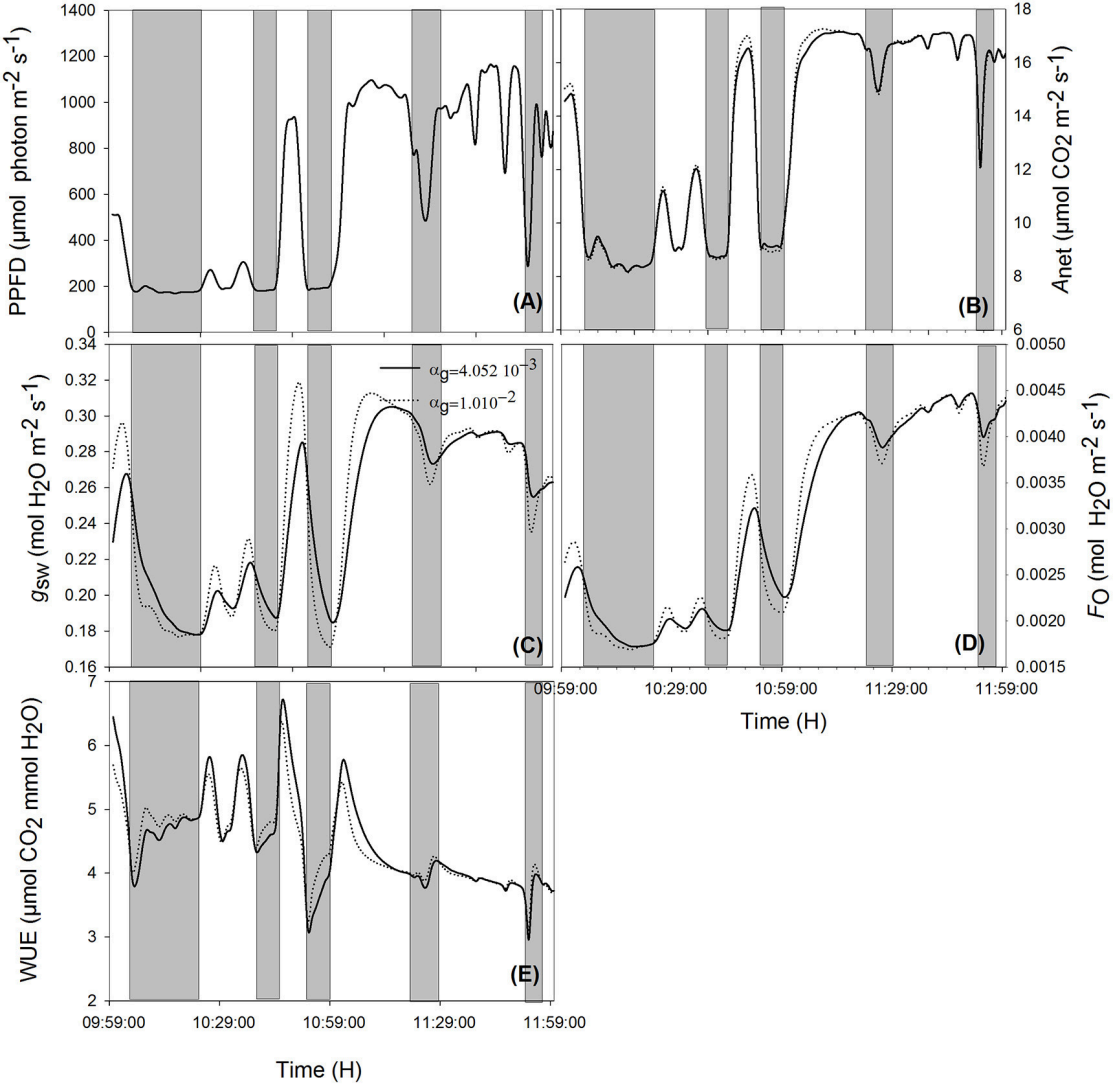
**Effect of varying time constant for stomata response (α_g_) on net assimilation rate (B), stomatal conductance (C), transpiration rate (D), water use efficiency (E) for different photosynthetic active radiation (PAR; A) during the sunny day**. Simulations were performed in steps of 1 s over 2 h (from 10:00 a.m. to 12:00 p.m.) and are extracted from measurements over 2 days data. Solid lines represent the fastest stomata opening/closing ($\alpha_{\text{g}}=4.052\cdot 10^{-3}$) and dotted lines represent the simulation result for $\alpha_{\text{g}}=1.01\cdot 10^{-2}$. Highlighted areas correspond to time courses where slower reacting stomata have a better water use efficiency. Other climatic data were extracted from the 2 days measurement data presented in Figure 1, and all other parameter values are defined in Tables 1, 2.

**Figure 6:**
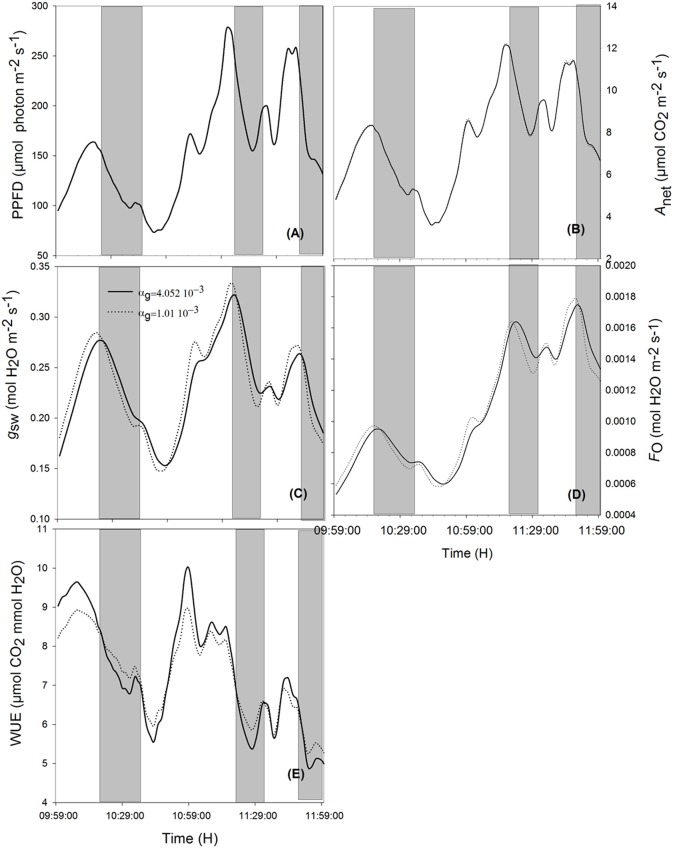
**Effects of varying the time constant for stomata response (α_*g*_) on net assimilation rate (B), stomatal conductance (C), transpiration rate (D), water use efficiency (E) for different photosynthetic active radiation (PAR; A) during the cloudy day**. Simulations were performed in steps of 1 s over 2 h (from 10:00 a.m. to 12:00 p.m.) and are extracted from measurements over 2 days' data. Solid lines represent the slowest stomata opening/closing (α_*g*_ = 4.052 10^−3^) and dotted lines represent the simulation result for α_g_ = 1.01 10^−2^. Shaded areas correspond to the period where slower reacting stomata have a higher water use efficiency. Other climatic data were extracted from the 2 days' measurement data presented in Figure 1 and all other parameter values are defined in Tables 1, 2.

When ambient light drops quickly, stomata with slower opening/closure time need more time to close, and, due to reduced radiation (Figures 5A, 6A, shaded area), the evaporation rate (Figures 5D, 6D, shaded area) is higher, and the stomatal limitation to net assimilation is lower (Figures 5B,C, 6B,C, shaded area). Therefore, WUE is lower for the slower reacting stomata (Figures 5E, 6E, shaded areas). The impact on WUE depends on climatic conditions on that day. In case of constant light intensity over the day, the stomatal speed did not affect WUE. A higher stomatal speed instantaneously increased WUE by up to 6.25% on cloudy day, depending on light variation (Figures 5E, 6E, shaded areas). The model was tested for different light exposure scenarios, including extreme cases when PPFD on sunny days was multiplied by 2, the temperature multiplied by 1.5 and the relative humidity multiplied by 0.6, and for different values of boundary layer conductance. Similar results were observed for all scenarios (Figures 5E, 6E).

### Effects of increasing maximum stomata conductance on WUE

**Figure 7:**
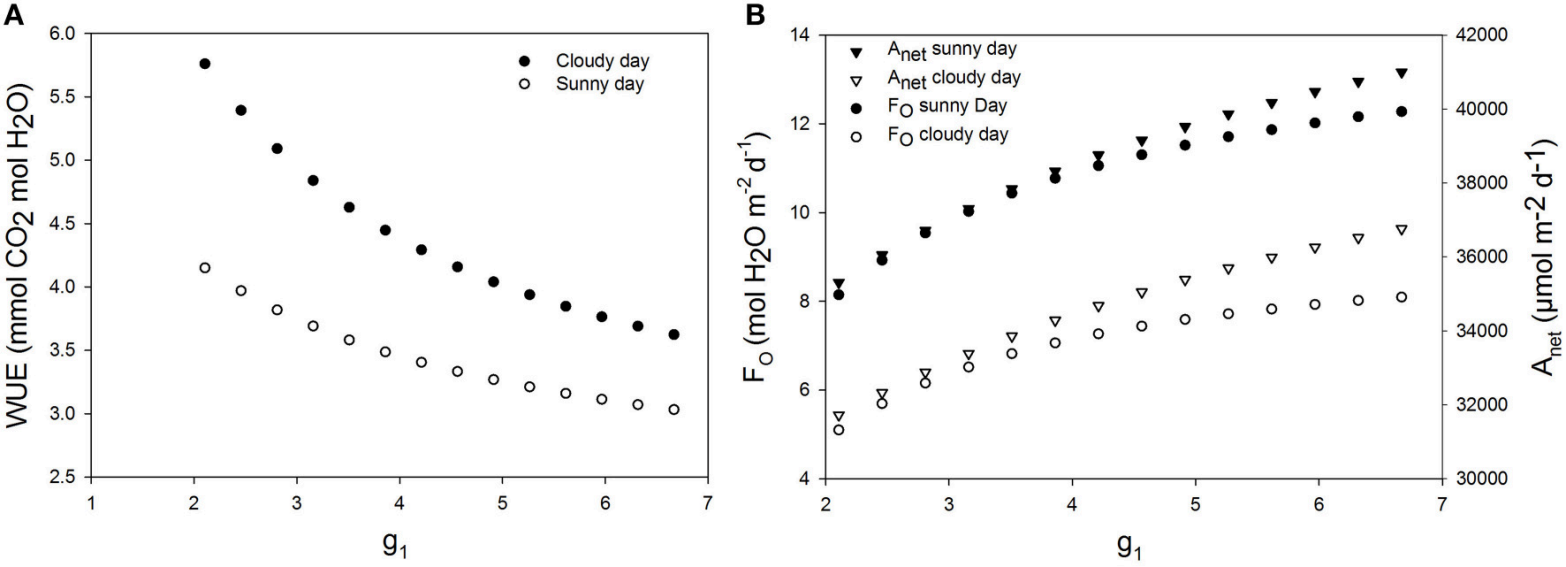
**Effect of increasing maximum stomatal conductance on daily water use efficiency. *g*_1_ is a parameter characterizing the maximum stomatal conductance**. **(A)** Daily WUE for different values of g_1_ for a cucumber leaf during the recorded sunny and cloudy days. **(B)** Total daily transpiration rate (*F*_*O*_) and daily net assimilation rate (*A*_net_) for different values of the maximum stomatal conductance. Simulations were performed per second over 15 h (from 06:00 a.m. to 9:00 p.m.). Values of *g*_1_= 3.51 was increased from −40 to 90% and each point on the figure is 1 day simulation result. Other climatic data were extracted from the 2 days measurement data presented in Figure 1 and all other parameter values are summarized in Tables 1, 2.

An increase of 20% of maximum stomatal conductance lead to a decrease of WUE of up to 8.66% during the sunny day and 8.57% during the cloudy day (Figure 7). In fact, increasing the maximum stomatal conductance increased the actual stomatal conductance and therefore transpiration rate. A higher net assimilation rate was also obtained in all cases, but the increased net assimilation did not compensate the water lost, and therefore, the WUE decreased for all stomatal aperture and closure speeds.

### Effects of higher mesophyll CO_2_ conductance on WUE

**Figure 8:**
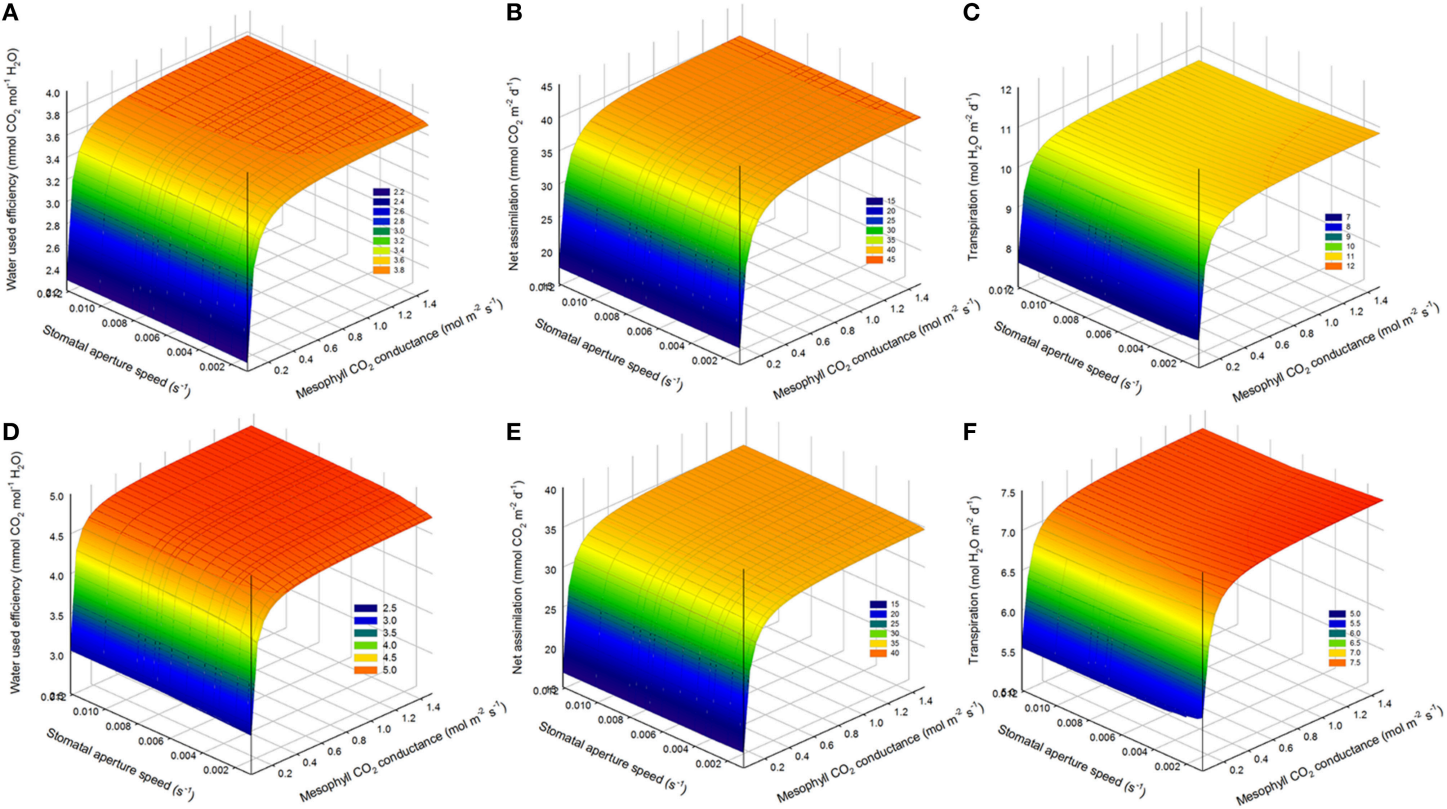
**Combined effect of increasing mesophyll CO_2_ conductance (*g*_m_) and stomatal speed (α_g_) on daily water use efficiency (WUE)**. **(A,D)** daily WUE for different values of *g*_m_ and **α**_g_ for a cucumber leaf during the recorded sunny **(A)** and cloudy **(D)** days. **(B,E)** total daily transpiration rate (*F*_O_) during the recorded sunny **(B)** and cloudy **(E)** days for different values of *g*_m_ and **α**_g_. **(C,F)** total daily net assimilation rate (*A*_net_) during the recorded sunny **(C)** and cloudy **(F)** days for different values of *g*_*m*_ and **α**_g_. Simulations were performed per second over 15 h (from 06:00 a.m. to 9:00 p.m.). Values of *g*_*m*_ = 0.3 was increased from −40 to 500% and **α**_g_ was varied in a set of 15 values and the figure present daily simulation result. Other climatic data were extracted from the 2 days measurement data presented in Figure 1 and all other parameter values are summarized in Tables 1, 2.

Increasing mesophyll CO_2_ conductance increased daily WUE by up to 4.5% on the cloudy day and 5.5% on the sunny day, despite an increase in transpiration rate (1.5%, Figures 8A,C,D,F). However, increasing *g*_m_ beyond 0.8 mol m^−2^ s^−1^, had only negligible effects on WUE for all stomatal speeds. Combining an increase in stomatal speed and *g*_m_ slightly increased WUE, and increased *A*_net_ more than the only effect of *g*_m_ (Figures 8B,E).

### Effects of fluctuating irradiance on WUE

**Figure 9:**
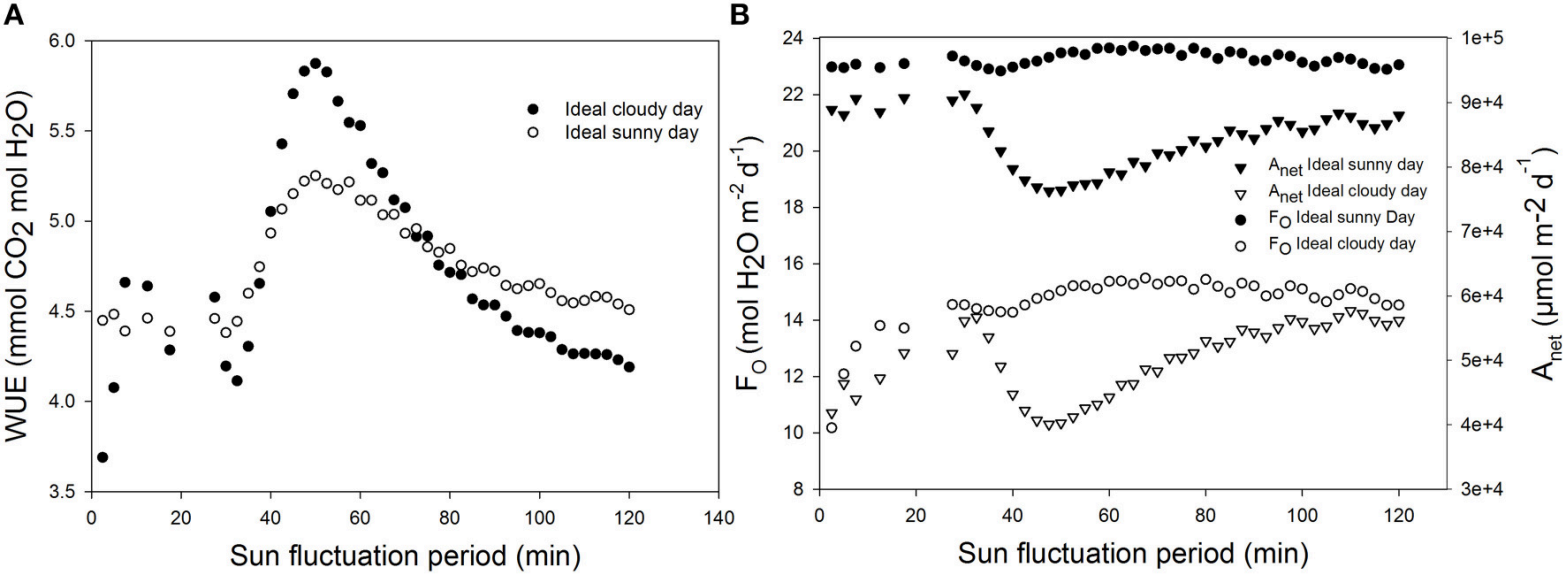
**Effect of increasing light and temperature fluctuation frequency on daily (A) water use efficiency (WUE), (B) total daily transpiration rate (F_O_), and daily net assimilation rate (*A*_net_)**. Simulations were performed per second over 15 h (from 06:00 a.m. to 9:00 p.m.). Value of the light sinusoidal period was increased from 1 min 25 s to 2 h and each point on the figure represents one day simulation summarized. All other parameter values are presented in Tables 1, 2.

Using ideal sunny day and ideal cloudy day scenarios, the light period was changed from 2 min 30 s to 2 h. The results show that WUE is maximal when the fluctuation period is around 60 min (Figure 9). WUE increased by as much as 70% depending on fluctuation period and total daily radiation.

## Discussion

Speed of stomatal response to environment, stomatal density and mesophyll conductance to CO_2_ have been proposed to be important traits of plant to better adapt to drought stress (Merlot et al., 2002; De Lucia et al., 2003; Warren et al., 2003; Büssis et al., 2006; Lawson and Blatt, 2014; Franks et al., 2015). Previous work reported that the speed of stomatal response might be more likely to enhance WUE than stomatal density (Lawson and Blatt, 2014), but their effects were not quantitatively assessed. To overcome the technical difficulties in quantifying the influence of these traits on daily WUE, a model describing the dynamics of WUE controlled by stomatal behavior and mesophyll CO_2_ conductance under different climatic conditions was presented.

### Stomatal speed

Although a faster reaction of stomatal conductance can increase the instantaneous WUE by up to 20% (Figures 5E, 6E), it did not improve daily WUE (Figures 4A,B). Fast stomatal opening speed in reaction to light increases carbon gain and water loss through transpiration at the same time. Therefore, it does not significantly increase WUE. The result is not in agreement with Lawson and Blatt (2014) who suggested that fast stomatal response increases both daily carbon gain and WUE. This discrepancy might be due to the fact that Lawson and Blatt (2014) calculated the intrinsic WUE (defined as *A*_net_/*g*_sw_) and ignored the influence of light energy on transpiration. Moreover, Lawson and Blatt (2014) argued that a slow stomatal response creates a stomatal limitation to photosynthesis and inferred that a fast response to light should reduce this limitation. However, the range of stomatal conductance from their measured data is 0.05–0.13 mol m^−2^s^−1^, a range where *g*_sw_ limitation would be strongest. In contrast, our *g*_sw_ data ranged from 0.15–0.30 mol m^−2^s^−1^, i.e., *g*_sw_ limitation was much less than in the dataset of Lawson and Blatt (2014). The model should be calibrated to plants with lower maximum *g*_sw_, to quantify the combined effect of α_g_ and *g*_1_ on WUE.

#### Effect of g_1_

Medlyn et al. (2011) demonstrated mathematically that the biological interpretation of *g*_1_ is WUE. This interpretation has been further proved by a global dataset showing that *g*_1_ reduces with available water in the soil (Lin et al., 2015). Therefore, it is not surprising that increase of *g*_1_ decreased WUE (Figure 7A). It is interesting to identify the traits determining *g*_1_. According to Equation (16), *g*_0_ and *g*_1_ are the physiological parameters which could increase *g*_sw_. Parameter *g*_0_ represents the *g*_sw_ value in the dark and is normally close to zero (although genotypes in *Arabidopsis* with constantly high *g*_sw_ in the dark have been found recently; Costa et al., 2015). Therefore, parameter *g*_1_ should be the factor determining the magnitude of *g*_sw_, which are related to stomatal size and density. This idea can be supported by the recent publication showing that reducing maximal *g*_sw_ by stomatal density increases WUE (Franks et al., 2015). Unfortunately, *g*_1_ was not estimated in this publication. Further, study which could show the relationship between *g*_1_ and stomatal density may help us to approach a more mechanistic understanding on *g*_1_. A minimum decrease of the daily WUE was 5.1% for an increase of *g*_1_ by 20%. This result does not agree with the instantaneous WUE measured by Franks et al. (2015) under steady state conditions. In contrast to Franks et al. (2015), our results suggest that increasing the maximum stomatal conductance will improve the net assimilation (more than 12.5%), but may not increase WUE. In fact, Franks et al. (2015) estimated a steady state WUE, by letting *g*_sw_ and *A* stabilize for 45 min, and therefore, did not account for the stomatal behavior under naturally changing environment. Under constant climatic conditions, a similar result was found (data not shown).

### Effect of light fluctuations

Increasing oscillation frequency of light and temperature could increase the WUE by up to 70%. The ideal oscillation period for a maximal WUE was found around 50 min. At this oscillation period, the stomatal responses and changes in light intensity may be synchronized in such a way that the light energy is optimally used by the leaf. In fact, stomatal guard cells react in response to changes in environmental conditions. If the light intensity reaches very fast a high value and drop quickly, then the stomatal aperture, because of the speed of response, may not reach the corresponding maximum target value. Therefore, the plant may not fully make use of the high light intensity due to a higher stomata limitation. This observation might explain results found in the literature. For example, the effects of environmental fluctuations on stomatal behavior were reported as reason for the limited effects of stomatal density (Lawson and Blatt, 2014). However, an increase of 70% in WUE for the same integral of light flux and temperature was unexpected. Further experiments may help to find an optimal light and temperature pattern leading to an optimal WUE for different plant species with different speed of stomatal response.

### Effects of mesophyll CO_2_ conductance

The model suggested that WUE can be improved by up to 5.5% by increasing mesophyll CO_2_conductance (*g*_m_). When *g*_m_ > 0.8 mol m^−2^ s^−1^, further increase in *g*_m_ did not significantly improve WUE. It might be explained by the fact that the ratio *A*_net_/*g*_m_ became very low in comparison with *C*_i_ for *g*_m_ > 0.8 so that the chloroplastic CO_2_ concentration and *C*_i_ are almost similar.

The model showed an influence of mesophyll CO_2_ conductance on E (Figure 8B). This could have been caused by the fact that the steady state target of stomatal conductance *G* is calculated from the estimated value of net *A*_net_ as modeled by Medlyn et al. (2011), which is a function of mesophyll CO_2_ conductance. The model can probably be improved by considering a different stomatal target model, independent of *A*_net_, but depending directly on environmental conditions (radiation, temperature, and vapor pressure deficit or relative humidity).

The model presented in this manuscript considered a fully expanded leaf and all photosynthetic parameters were taken constant during the simulation period. The effect of Rubisco activation and deactivation (Gross et al., 1991), and the age effect on photosynthetic parameters might allow a wider application of the model. Another issue for further development of the model is the canopy WUE, taking into account the effect of leaf age and canopy architecture.

## Conclusion

The model presented in this manuscript allowed to quantify the effects of stomatal behavior and mesophyll CO_2_ conductance on the WUE of a cucumber leaf. Combining stomatal dynamics with the effects of changing climatic condition on photosynthesis and transpiration rates allows to find that for the case of cucumber leaves that was analyzed, an increase of stomatal speed will not lead to an increase of more than 1.5% of the daily WUE under normal fluctuating light condition. It was also found that increasing maximum stomatal conductance decrease WUE. Increasing mesophyll CO_2_ conductance can lead to an increase of daily WUE by up to 5.5%. This suggests that increasing mesophyll CO_2_ conductance might be more likely to increase WUE than increasing stomatal density and speed.

## Author contributions

DM and TC Developed the model, DM performed all numerical simulations, DM, TC, and HS discussed the data and wrote the paper.

### Conflict of interest statement

The authors declare that the research was conducted in the absence of any commercial or financial relationships that could be construed as a potential conflict of interest.
